# Cinematic Rendering of Brain Magnetic Resonance Imaging: Photorealistic Visualization of Normal Neuroanatomy and Primary Brain Tumors

**DOI:** 10.5152/eurasianjmed.2026.261605

**Published:** 2026-09-01

**Authors:** Zeynep Fırat, Füsun Er, Antonio Di Ieva, Gazanfer Ekinci, Uğur Türe

**Affiliations:** 1 Department of Radiology, Yeditepe University Hospitals, İstanbul, Türkiye; 2 Department of Computer Engineering, Piri Reis University, İstanbul, Türkiye; 3 Computational NeuroSurgery (CNS) Lab, Macquarie Medical School, Macquarie University Faculty of Medicine and Health Sciences, Sydney, NSW, Australia; 4 Department of Neurosurgery, Yeditepe University Hospitals, İstanbul, Türkiye

**Keywords:** Brain MRI, cinematic rendering, medical education, neuroanatomy, 3-dimensional visualization

## Abstract

**Background:**

Cinematic rendering (CR) is an advanced post-processing technique that generates volumetric 3-dimensional (3D) visualizations from routine clinical magnetic resonance imaging (MRI) data, potentially enhancing anatomical interpretation and spatial understanding.

**Methods:**

In this retrospective single-center study, 3.0-T MRI datasets from 35 participants (15 healthy controls and 20 patients with histopathologically confirmed primary brain tumors) were analyzed. Cinematic rendering images were generated from precontrast 3D T1-weighted magnetization-prepared rapid acquisition gradient echo (MPRAGE) sequences. Two independent senior observers evaluated image resolution, anatomical detail, lesion depiction, visualization utility, and educational value using a 3-point Likert-type scale. Interobserver agreement was assessed using quadratic weighted Cohen’s *κ* with bootstrap-derived 95% confidence intervals. Statistical analyses were performed using IBM SPSS Statistics for Windows, Version 29.0 (IBM Corp.; Armonk, NY, USA).

**Results:**

Cinematic rendering provided consistent, high-fidelity 3D visualization of normal neuroanatomy and tumor-related structural alterations across different primary brain tumor histologies. Image quality ratings were uniformly high (median score: 3), with no significant differences between groups (*P* ≥ .229). Educational value was rated consistently by both observers across all cases. Interobserver agreement ranged from substantial to almost perfect (*κ* = 0.65-1.00), with perfect agreement for lesion depiction (*κ* = 1.00; 100% agreement).

**Conclusion:**

Cinematic rendering applied to routine brain MRI provides reproducible, depth-enhanced 3D visualization of normal brain anatomy and tumor-related spatial relationships. The technique demonstrates consistent image quality and interpretability across different tumor types and may serve as a supplementary tool for neuroanatomical education and spatial understanding in clinical practice.

**Main Points**

- Cinematic rendering enables detailed 3-dimensional (3D) visualization of normal brain anatomy using routine 3D T1-weighted MRI.
- It provides accessible and practical 3D visualization of the living brain without requiring additional imaging protocols.
- It improves the depiction of spatial relationships between brain tumors and surrounding neuroanatomical structures.
- Image quality and interpretability are consistently high across different primary brain tumor histologies.
- Cinematic rendering may be useful as a supplementary tool for anatomical education and spatial orientation in selected clinical settings.

## Introduction

Accurate visualization of brain anatomy is fundamental to medical education and neuroscience training; however, interpreting complex 3-dimensional (3D) anatomical relationships from conventional 2-dimensional (2D) MRI remains a recognized challenge.^1^^-^^3^ Slice-based image interpretation may limit depth perception and spatial understanding of cortical and subcortical structures, and these limitations are amplified in educational settings where learners must translate 2D representations into meaningful 3D conceptual models.^4^^,^^5^ This has increased interest in visualization approaches capable of conveying neuroanatomical relationships more intuitively, particularly as digital tools increasingly complement traditional teaching methods.^6^^,^^7^

Cinematic rendering (CR) is an advanced post-processing technique that applies physically based global illumination algorithms to volumetric imaging datasets, generating 3D images with enhanced depth perception and surface detail.^6^^-^^10^ Unlike conventional volume rendering, which relies on ray casting and simplified opacity transfer functions, CR simulates the physical interaction of light with tissue, producing shadows, reflections, and surface gradients that closely approximate the visual experience of examining real anatomical specimens.^6^^,^^8^^,^^11^ To date, CR has been studied predominantly in computed tomography (CT) and extracranial applications, where it has demonstrated superior anatomical representation compared to conventional volume rendering.^12^^-^^14^ Similarly, efforts to enhance neuroanatomy education through digital tools have focused largely on virtual reality environments and computer-generated 3D atlases,^14^ which, while effective, rely on synthetic models rather than real patient data. In contrast, the application of CR to brain MRI remains largely unexplored, despite MRI being the principal modality in neuroimaging^15^^-^^17^ and offering the tissue contrast and spatial resolution necessary for high-fidelity reconstruction. Recent extensions of CR to MRI data beyond the brain, including peripheral nerve and musculoskeletal imaging,^9^^,^^18^ further support the versatility of the technique with volumetric MRI datasets. High-resolution 3D T1-weighted sequences, routinely acquired in clinical practice, provide the anatomical detail and gray–white matter contrast required for CR,^16^^,^^17^^,^^19^ representing an accessible substrate for patient-derived educational visualization without additional imaging burden.

A distinctive feature of MRI-based CR is its capacity to illustrate 2 complementary educational dimensions within a single dataset: in healthy individuals, it conveys sulcal-gyral patterns and cortical morphology with volumetric realism; in pathological cases, it renders the 3D consequences of disease on brain parenchyma, including tumor displacement, infiltration, and cortical deformation. Both dimensions are educationally relevant and rarely available together in a single, reproducible format derived from real clinical data.

To date, systematic evaluation of MRI-based CR as an educational resource in neuroanatomy is lacking. While virtual reality and computer-generated anatomical models have been investigated for spatial learning,^14^ and CR has been applied to CT-based neuroimaging for visualization and pre-therapeutic purposes,^5^^,^^12^^-^^15^^,^^18^ a reproducibility-focused assessment of CR applied to brain MRI specifically for educational visualization has not been reported. The impetus for this study arose from clinical observations suggesting that CR applied to routine brain MRI offered enhanced spatial clarity in depicting neuroanatomical relationships and tumor-parenchyma interactions. Accordingly, this retrospective study aimed to evaluate the reproducibility and educational utility of CR-generated visualizations from routine 3D T1-weighted brain MRI in both healthy individuals and patients with primary brain tumors, using a blinded, multi-observer design with a standardized scoring framework, and to provide a reproducible methodological basis for future learner-centered investigations.

## Material and Methods

### Study Design and Participants

This retrospective, single-center observer study was conducted in accordance with the principles of the Declaration of Helsinki; informed consent was waived due to the retrospective nature of the study.

Between January and November 2024, 35 participants were enrolled: 15 healthy controls (no relevant neurological or psychiatric history, normal conventional MRI findings) and 20 patients with histopathologically confirmed, newly diagnosed primary brain tumors (no prior cranial surgery). Five histopathological subtypes were equally represented (n = 4 each): dysembryoplastic neuroepithelial tumor (DNET), astrocytoma, oligodendroglioma, glioblastoma, and meningioma (Table 1). This deliberate selection spanned the biological spectrum, ranging from low-grade cortical lesions to infiltrative high-grade tumors and extra-axial masses, and was intended to assess the consistency of CR’s educational utility across pathologies with markedly different anatomical presentations. Case selection followed predefined inclusion criteria: histopathologically confirmed, newly diagnosed primary brain tumor with no prior cranial surgery, and availability of a high-quality precontrast 3D T1-weighted MPRAGE dataset meeting technical adequacy criteria for CR reconstruction (absence of severe motion artifacts, complete brain coverage). The 5 tumor subtypes were selected to represent distinct anatomical and pathological categories, cortical/subcortical low-grade lesions (DNET, oligodendroglioma, and astrocytoma), infiltrative high-grade parenchymal tumors (glioblastoma), and extra-axial masses (meningioma), thereby enabling assessment of CR performance across the widest feasible range of spatial distortion patterns within this pilot cohort.

Only examinations including adequate noncontrast, high-resolution 3D T1-weighted datasets suitable for CR reconstruction were included. Exclusion criteria comprised severe motion artifacts, incomplete brain coverage, or technical limitations precluding reliable reconstruction. Statistical analyses and observer evaluations were performed exclusively on CR images derived from precontrast 3D T1-weighted MRI; postcontrast images were generated solely for selected illustrative figures.

### Magnetic Resonance Imaging Acquisition

All examinations were performed on a 3.0-T system with a 48-channel head coil. High-resolution whole-brain structural imaging was acquired in the sagittal plane using precontrast and postcontrast 3D T1-weighted MPRAGE with the following parameters: TR/TE/TI = 2700/3.7/1000 ms; isotropic voxel size, 1 × 1 × 1 mm; matrix, 300 × 256; field of view, 240 mm.

### Cinematic Rendering

CR is a volumetric post-processing technique that applies physically based global illumination models to imaging data, simulating light–tissue interactions to generate photorealistic visualizations with enhanced depth and surface texture cues.^15^^,^^16^ Image appearance is governed by intensity-based transfer functions that map voxel intensities to defined tissue ranges, enabling selective suppression of background signal while emphasizing relevant anatomical boundaries.^15^^,^^20^

CR reconstructions were generated from precontrast 3D T1-weighted datasets using a dedicated cinematic rendering workstation running the Siemens Syngo.via platform with the Cinematic Rendering application (Siemens Healthineers, Erlangen, Germany). A single neuroimaging specialist performed all reconstructions using a standardized, 3-level thresholding protocol, calibrated to optimize (1) sulcal-gyral surface definition, (2) cortical morphology and gyral surface detail, and (3) cortical-subcortical structural boundaries. These threshold levels were established against a reference anatomical template prior to data analysis and applied uniformly across all cases. Opacity transfer functions were adjusted within each threshold level to selectively suppress background signal while preserving tissue-boundary contrast. The entire CR reconstruction workflow per case, including image transfer, threshold adjustment, and export, was completed within approximately 3-5 min using the routine clinical 3D T1-weighted MPRAGE dataset, without requiring additional imaging acquisitions. Finalized CR image sets were exported with identical display settings and provided to both observers for independent blinded evaluation.

### Visual Scoring and Observer Assessment

CR datasets were independently evaluated by 2 senior neuroimaging specialists, each with over 20 years of experience in neuroimaging. Observers were blinded to clinical data, imaging parameters, and group allocation; cases were presented in randomized, mixed order to mitigate recall bias. It should be acknowledged, however, that complete blinding to the presence of pathology was not always achievable, as tumor-related structural distortion may be visually apparent on CR images. Randomization and mixed presentation were therefore employed to reduce, rather than eliminate, the potential for performance bias in this perceptual evaluation framework.

The assessment framework was designed to measure visualization fidelity and perceived educational utility rather than diagnostic performance. Expert observer evaluation was selected as the appropriate methodology for this preliminary feasibility study, as the primary aim was to establish whether CR-generated images meet the technical and perceptual standards required for educational use, a judgment that requires domain expertise to assess reliably. Formal learner-centered evaluation, which would be necessary to demonstrate actual pedagogical impact, is outside the scope of this initial reproducibility-focused study and is identified as a priority for future research. Five predefined domains were evaluated: (1) Image Resolution, defined as sharpness and clarity of the reconstructed volume; (2) Anatomical Detail, defined as visibility of specific neuroanatomical landmarks; (3) Lesion Depiction (tumor cases only), defined as clarity of the tumor-parenchyma interface; (4) Visualization Utility, defined as the overall effectiveness of the 3D representation for depicting spatial relationships; and (5) Educational Value, defined as the perceived suitability of the images for anatomical instruction and illustration. Ratings were recorded on a 3-point Likert-type scale, intentionally simplified to enhance interobserver consistency in a perceptual assessment framework. The scoring criteria for each grade are summarized in Table 2.

### Statistical Analysis

Group comparisons between healthy controls and tumor patients were performed using the Mann–Whitney *U-*test. Observer-averaged scores were calculated as the median of 2 independent ratings per case and used in exploratory analyses assessing associations with age (dichotomized at 40 years), sex, lesion size (median split), and anatomical location. The Benjamini–Hochberg false discovery rate (FDR) correction was applied to control for multiple comparisons, with adjusted *q*-values reported where applicable. Non-parametric effect sizes were expressed as *r*-values. Interobserver agreement was assessed using quadratic weighted Cohen’s *κ* with 95% confidence intervals derived from bootstrapping (1000 iterations). Statistical analyses were performed using IBM SPSS Statistics for Windows, Version 29.0 (IBM Corp.; Armonk, NY, USA). Statistical significance was set at *P* (or *q*) < .05.

This retrospective study was approved by the Institutional Clinical Research Ethics Committee of Yeditepe University (Approval No: 202311Y0699; Date: December 13, 2024), and the requirement for informed consent was waived due to the retrospective study design.

## Results

### Participant Characteristics

The study cohort comprised 35 participants (mean age 37.4 ± 16.3 years; 51.4% female). The healthy control group (n = 15) had a mean age of 35.6 ± 17.85 years, and the tumor group (n = 20) a mean age of 38.8 ± 15.35 years. The tumor cohort showed an even histopathological distribution (n = 4 each: DNET, oligodendroglioma, astrocytoma, glioblastoma, and meningioma), with the temporal lobe as the most frequent site (n = 10) and a broad range of lesion dimensions (12 × 10 × 11 mm to 67 × 63 × 45 mm) (Table 1).

### Visual Quality and Educational Value Ratings

**Figure 1:**
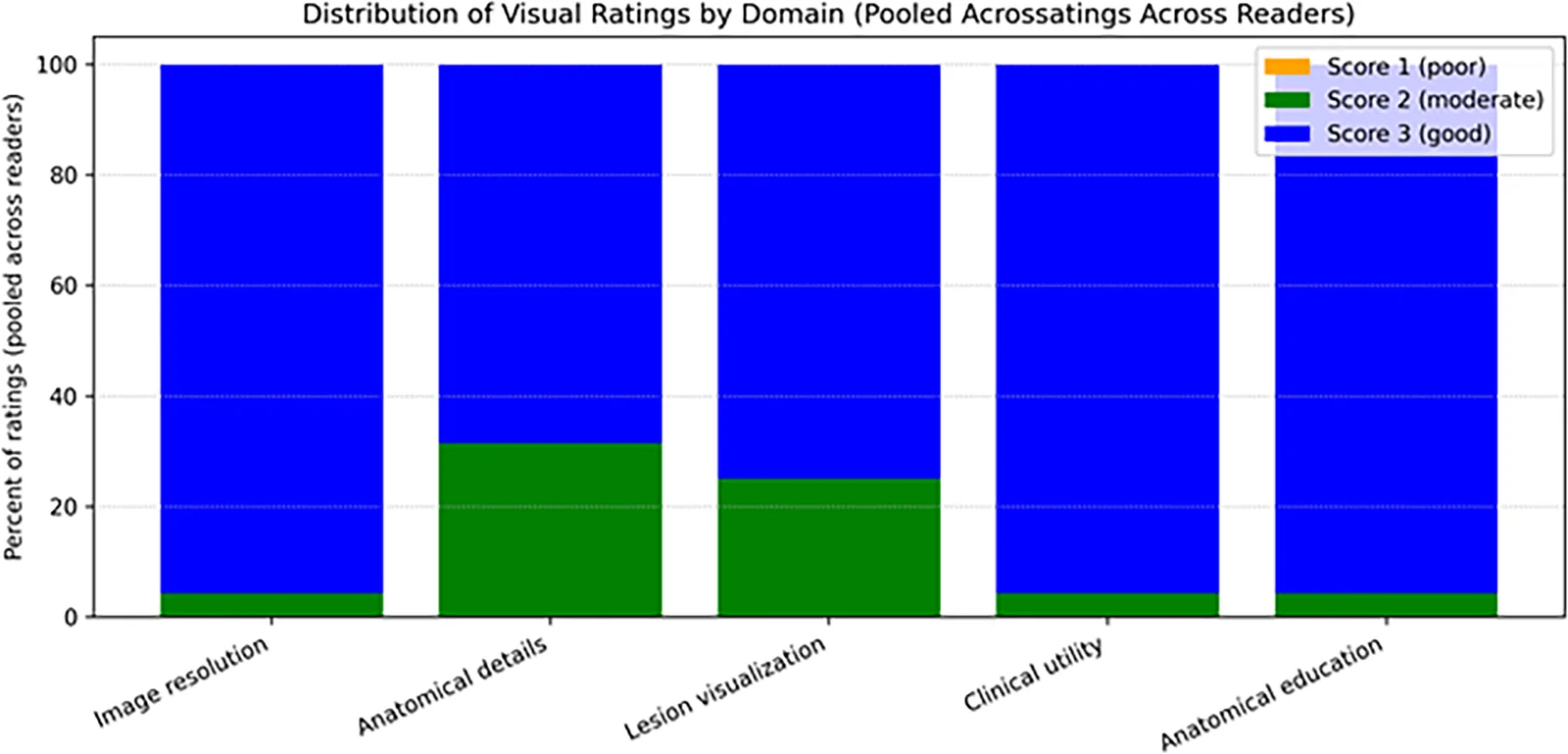
Distribution of visual quality and educational value ratings across all evaluation domains. Stacked bar charts display the proportion of 3-point Likert ratings (1 = poor, 2 = moderate, 3 = good) pooled across both independent observers for healthy controls (n = 15) and brain tumor cases (n = 20). The uniformly high rating frequency (Grade 3) across all domains, including educational value, and the absence of significant group differences illustrate the visual consistency of CR-based representations irrespective of underlying pathology, supporting their reliability as standardized educational resources.

CR consistently generated high-quality, visually detailed 3D representations across all cases. Median visual quality ratings were uniformly 3 (“Good”) for both observers in both groups across all evaluated domains. Comparative analysis revealed no statistically significant differences in any domain between healthy controls and tumor patients (*P* ≥ .229 for all comparisons, Table 3; Figure 1). This consistency is particularly notable for the educational value domain, where both observers assigned identical scores across all 35 cases regardless of the presence or histopathological type of tumor (Median: 3 [IQR : 3-3] for both groups and both observers). The finding that pathological distortion, including mass effect, infiltration, and cortical displacement, did not reduce the perceived educational utility of CR images indicates the robustness of the technique as a teaching resource across a wide range of neuroanatomical states.

### Interobserver Reliability

**Figure 2:**
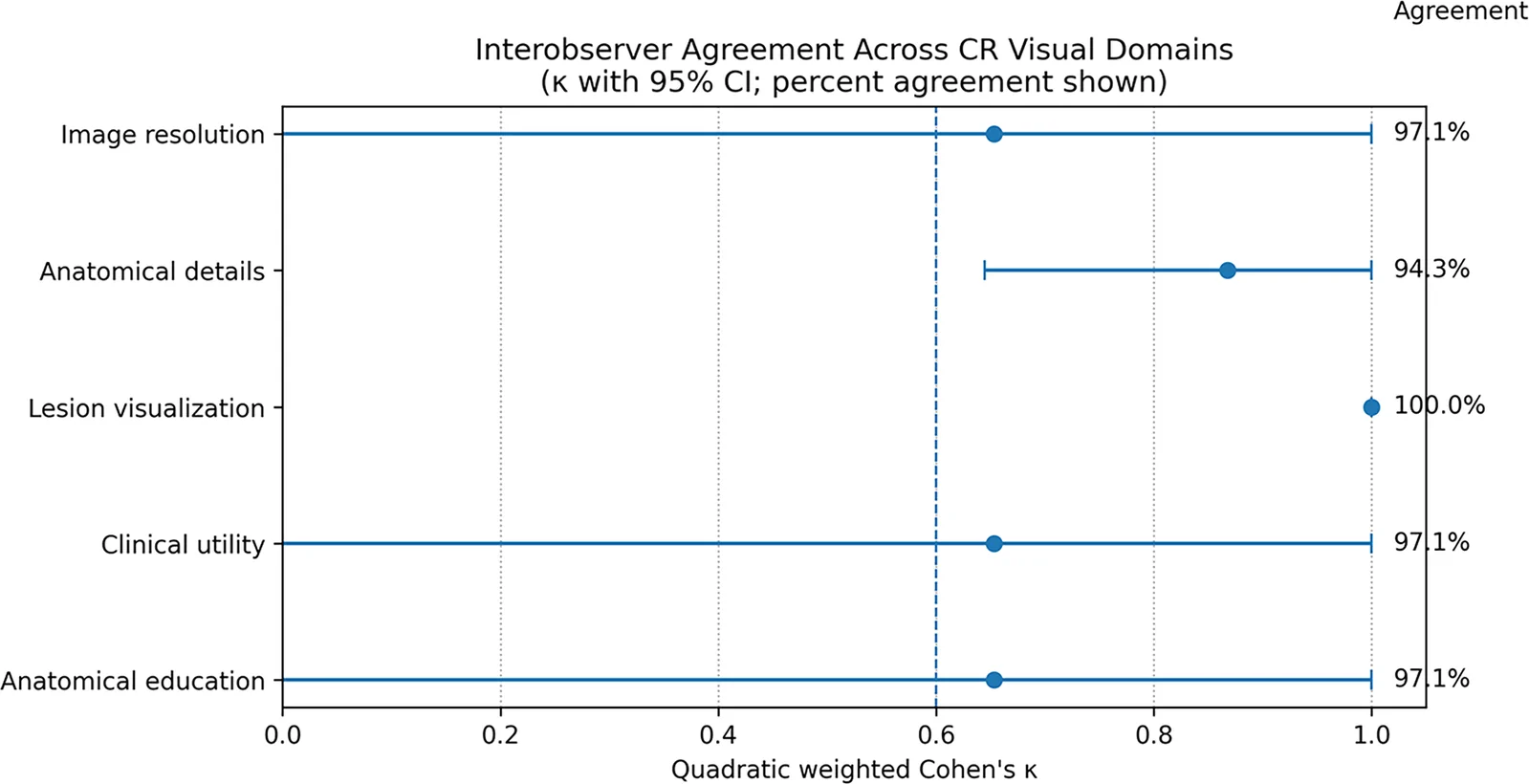
Interobserver agreement for cinematic rendering (CR) visual domains. Forest plot displaying quadratic weighted Cohen’s *κ* values with 95% bootstrap-derived confidence intervals (1000 iterations) and absolute percent agreement rates for all evaluated domains. The absolute consensus for lesion depiction (*κ* = 1.00, percent agreement: 100%) and near-ceiling percent agreement across all domains (94.3%-100%) confirm the high reproducibility of CR-based assessments, a prerequisite for consistent deployment as an educational tool.

Measures of interobserver agreement confirmed robust reproducibility across all evaluated domains (Table 4, Figure 2). Quadratic weighted Cohen’s *κ* demonstrated almost perfect agreement for anatomical detail (*κ* = 0.87, 95% CI: 0.64-1.00) and absolute consensus for lesion depiction in the tumor subgroup (*κ* = 1.00, 95% CI: 1.00-1.00; percent agreement: 100%). For image resolution, visualization utility, and educational value, substantial agreement was observed (*κ* = 0.65 for each), with high percent agreement rates of 94.3%-97.1%. The lower *κ* values for these 3 domains reflect a “ceiling effect” attributable to the high frequency of maximum scores rather than true observer disagreement, a pattern consistent with the near-universal perception of high-quality imaging output.

### Demographic and Lesion-Related Associations

Exploratory analysis indicated that visual quality and educational utility ratings were largely independent of participant demographic characteristics. Although preliminary comparisons suggested minor age-related differences in anatomical detail and lesion visualization scores within the tumor group (*P* = .034 and .026, respectively), these associations did not survive FDR correction (both *q* = 0.084). No significant associations were identified for sex (*q* = 0.967). Lesion size and anatomical location did not significantly influence ratings in any domain, suggesting that the educational utility of CR is not contingent on lesion characteristics.

### Illustrative Visualization Examples

**Figure 3:**
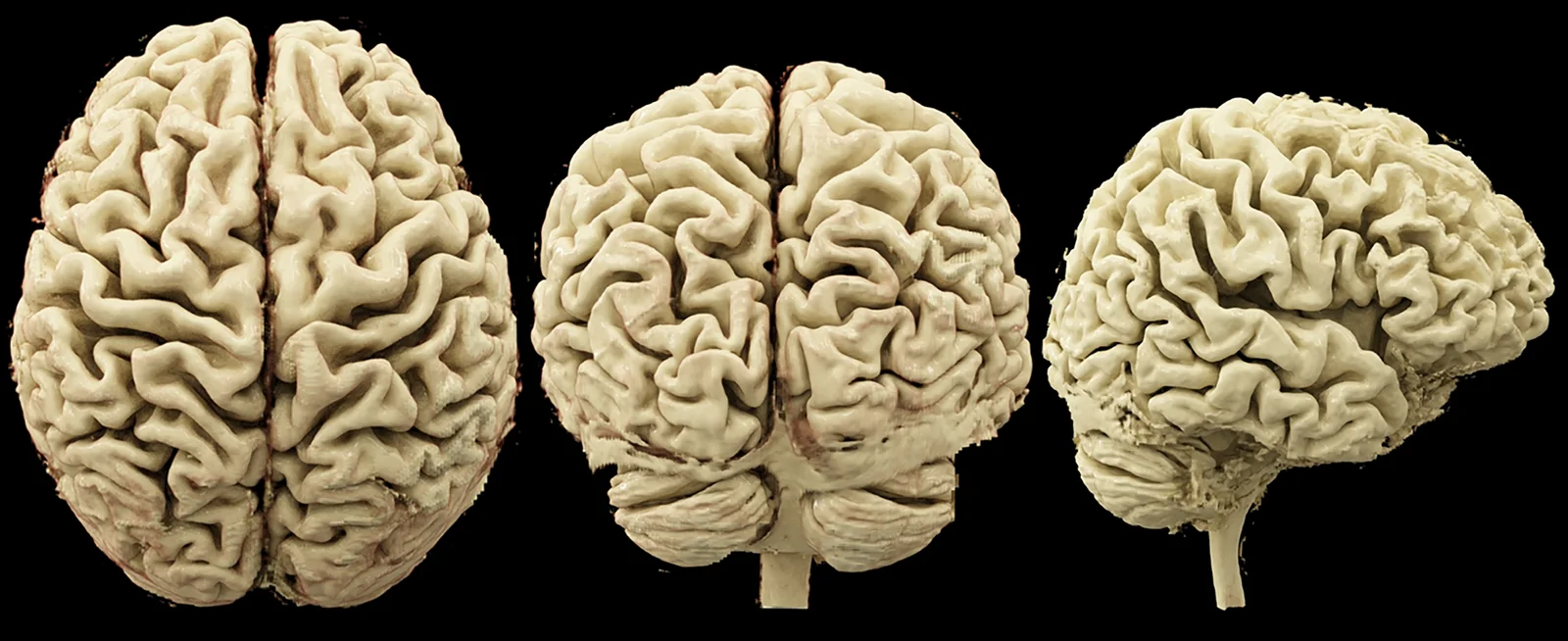
Photorealistic 3D visualization of normal neuroanatomy in a healthy volunteer. Axial, coronal, and sagittal CR reconstructions from an 85-year-old participant demonstrate the detailed, high-fidelity representation of cortical morphology and sulcal–gyral architecture achievable from routine precontrast 3D T1-weighted MRI. These images illustrate the capacity of CR to present individual anatomical variability and fine surface detail in a format that closely approximates the visual experience of examining a real brain specimen, providing an in vivo, patient-specific complement to conventional anatomical teaching resources.

The quantitative findings were complemented by a set of representative CR visualizations selected to demonstrate the educational range of the technique. In healthy controls, CR produced detailed, depth-resolved reconstructions of the brain surface, revealing sulcal-gyral architecture, cortical folding patterns, and interhemispheric relationships with a surface realism not achievable through conventional 2D imaging (Figure 3). These images illustrate the capacity of routine MRI data, when processed through CR, to serve as a high-fidelity reference for normal neuroanatomical instruction.

**Figure 4:**
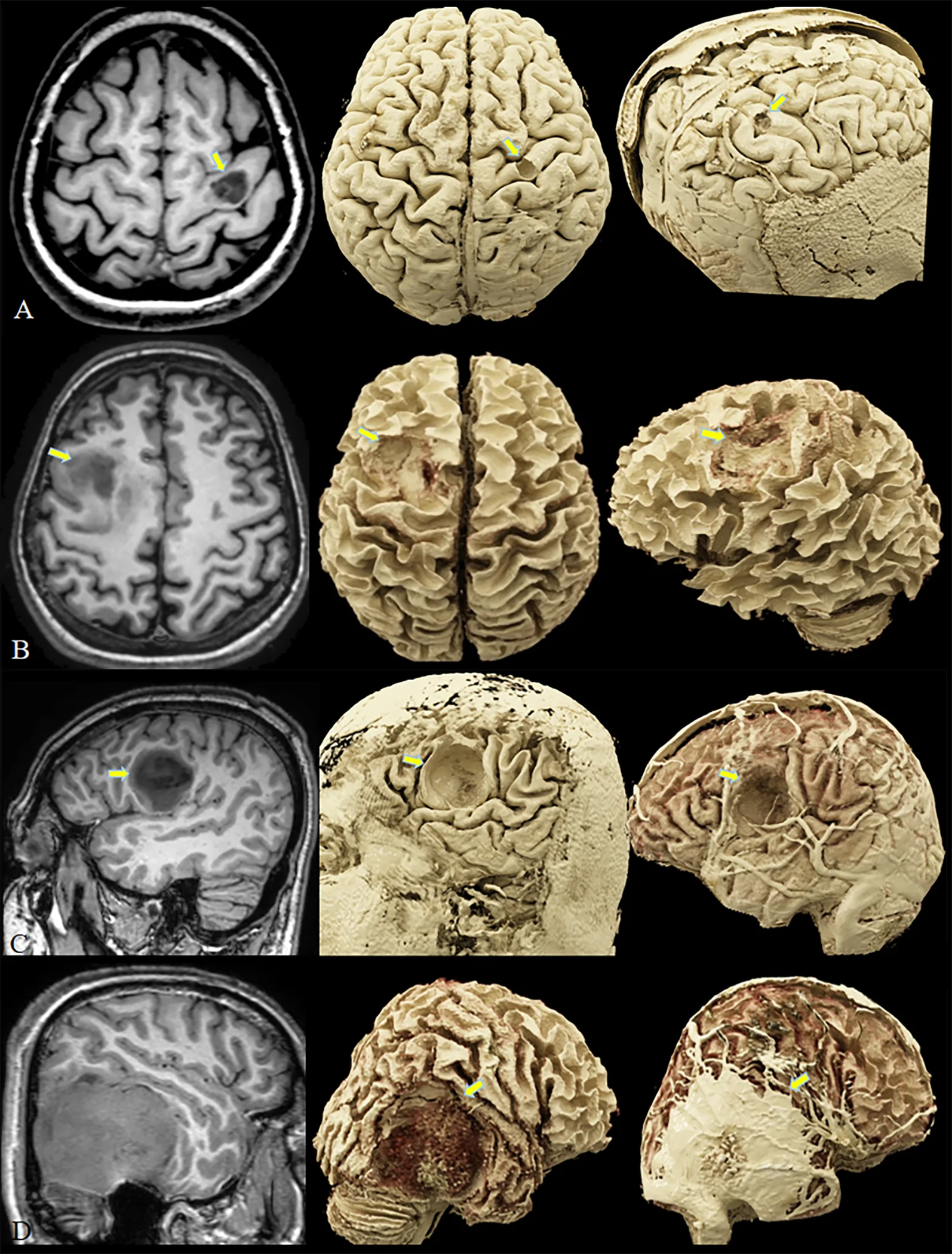
Cinematic rendering across primary brain tumor subtypes: spatial impact of pathology on neuroanatomy. Yellow arrows indicate the primary lesion. Each panel illustrates the characteristic 3-dimensional anatomical distortion pattern of each tumor subtype. (A) DNET (38-year-old): right temporal cortical lesion with well-demarcated gyral expansion and cortical thickening on axial and sagittal CR. (B) Oligodendroglioma (34-year-old): infiltrative frontal lesion with broad cortical involvement and sulcal effacement on axial CR. (C) Glioblastoma (54-year-old): precontrast CR shows extensive mass effect and cortical displacement; postcontrast CR depicts heterogeneous enhancing rim and intralesional vascularity. (D) Meningioma (49-year-old): right temporal extra-axial mass with hemispheric displacement on sagittal CR; postcontrast CR demonstrates dural attachment and homogeneous enhancement.

In tumor cases, CR consistently depicted the 3D spatial consequences of each pathology on the surrounding brain parenchyma in a visually intuitive format (Figure 4). The cortical expansion and gyral distortion of DNET, the broad infiltrative involvement of oligodendroglioma, the complex intralesional architecture and mass effect of glioblastoma, and the extra-axial displacement and dural attachment of meningioma were each rendered with consistent 3D spatial clarity across pre- and postcontrast acquisitions. These illustrative examples demonstrate that CR can convey, within a single standardized visualization format, both the characteristic appearance of each tumor subtype and its specific impact on adjacent neuroanatomy, supporting its potential utility as a demonstrative educational resource across a biologically diverse spectrum of brain pathologies.

## Discussion

This study demonstrates that volumetric cinematic rendering can be reliably and reproducibly generated from routine 3D T1-weighted brain MRI, maintaining consistently high visual quality across both normal neuroanatomy and a spectrum of primary brain tumors. Educational value was rated as uniformly high by 2 independent senior observers regardless of pathological complexity, histopathological subtype, lesion size, or patient demographics. These findings support cinematic rendering as a technically feasible and reproducible visualization approach with perceived educational potential, applicable to routinely acquired clinical MRI data. It should be noted that this study evaluates perceptual quality and expert-rated suitability for educational purposes; learner-centered validation through formal educational studies will be required to confirm the practical pedagogical benefit of this approach.

The primary educational value of cinematic rendering lies in its ability to generate patient-derived, in vivo 3D visualizations that differ fundamentally from conventional teaching resources. Unlike synthetic anatomical models, cinematic rendering preserves individual anatomical variability, intrinsic tissue contrast, and the true spatial effects of pathology within a living anatomical environment. In healthy subjects (Figure 3), this approach provides detailed visualization of cortical and subcortical anatomy with a depth and surface realism that may help bridge the gap between schematic representations and real anatomical structures. Prior studies have shown that 3D visualization tools, including virtual reality and digital anatomy platforms, improve spatial understanding and anatomical learning in medical education.^14^^,^^19^^,^^20^ In this context, cinematic rendering represents a complementary approach, as it is directly derived from routine clinical imaging rather than synthetic or cadaver-based models, thereby integrating anatomical education with real patient data. Importantly, a direct head-to-head comparison with VR or other visualization modalities was not the aim of this study; the existing literature already documents VR-based and atlas-based approaches in neuroanatomy education, and the present work was designed to establish the reproducibility and perceptual foundation of MRI-based CR specifically in the neuroanatomical domain, a context in which such a systematic evaluation had not previously been reported.

The present study builds on earlier work by Lakhani and Deib,^16^ who first demonstrated the feasibility of cinematic rendering in brain MRI for qualitative assessment of intracranial tumors and spatial relationships. Their study highlighted the potential of CR for pretherapeutic visualization; however, it did not include a structured educational assessment or a healthy control group. The current study extends these findings by introducing a reproducibility-focused and education-oriented evaluation framework, incorporating both normal neuroanatomy and multiple tumor histologies, as well as formal interobserver agreement analysis. In addition, prior cinematic rendering studies have predominantly focused on CT-based applications and extracranial anatomy,^12^^-^^14^ whereas MRI-based applications remain limited.

The diversity of tumor histologies included in this dataset adds an additional educational dimension (Figure 4). Each tumor type produces distinct patterns of spatial distortion, and understanding these 3D relationships is essential for both neuroanatomy education and neuro-oncological reasoning. The consistently high lesion depiction scores, with perfect interobserver agreement (*κ* = 1.00), suggest that cinematic rendering reliably conveys these spatial relationships in a clear and interpretable manner. From an educational perspective, this allows learners to intuitively appreciate how different tumor types alter brain anatomy in 3 dimensions, which is often less apparent on conventional 2-dimensional imaging.

A key advantage of MRI-based cinematic rendering is its accessibility. The technique is generated as a post-processing application of routinely acquired 3D T1-weighted MPRAGE sequences and does not require additional imaging protocols or dedicated acquisitions beyond standard clinical practice. As such, institutions with standard 3.0-T MRI systems and compatible software can potentially generate high-quality educational datasets from existing clinical images. Prior work in anatomy education has similarly demonstrated that CR-based resources integrated into teaching settings are associated with high learner satisfaction and improved spatial awareness,^20^ supporting its feasibility as an educational adjunct.

Several practical considerations relevant to the broader implementation of MRI-based CR merit discussion. First, with respect to computational requirements, the Siemens Syngo.via platform used in this study completed the full reconstruction workflow within approximately 3-5 min per case, representing a clinically feasible processing burden that does not require dedicated high-performance computing infrastructure beyond a compatible workstation. Second, although CR is generally robust for high-resolution isotropic datasets, the technique is susceptible to certain image quality limitations: motion artifacts, susceptibility-related distortion, and regions of low tissue contrast may degrade reconstruction quality and produce surface rendering artifacts that could reduce anatomical clarity. These artifacts were managed in the present study through predefined inclusion criteria that excluded examinations with severe motion or technical inadequacies; however, their potential impact in routine, unselected clinical datasets should be recognized. Third, workflow integration requires compatible post-processing software and institutional access to the relevant platform; currently, CR availability remains limited to institutions with the appropriate vendor-specific infrastructure, which may restrict its immediate scalability as a routine educational resource. Addressing these implementation barriers will be important for future studies seeking to deploy CR-based educational frameworks across heterogeneous institutional environments.

The reproducibility findings of this study further support its potential educational utility. Consistent interpretation across observers is essential for any imaging-based teaching resource. The substantial to almost perfect interobserver agreement observed across all evaluation domains (*κ* = 0.65-1.00; 94.3%-100% agreement) indicates that cinematic rendering provides stable and reproducible visual outputs that are interpreted similarly by expert readers. The near-universal high scoring observed in several domains likely reflects a ceiling effect, suggesting consistently high perceived image quality rather than variability in interpretation. Previous comparative studies have also shown that cinematic rendering facilitates rapid and consistent interpretation of anatomical structures compared with conventional imaging techniques.^21^

The consistently high educational ratings across both normal and pathological cases suggest that cinematic rendering may function as a unified visual resource for neuroanatomy education. In particular, the combination of healthy brain datasets and tumor cases allows simultaneous teaching of normal anatomy and pathology-induced spatial alterations within a single framework. Within the broader context of digital anatomy education, cinematic rendering occupies a complementary position alongside virtual reality, augmented reality, and other immersive visualization technologies.^14^^,^^19^^,^^20^ Early studies have also demonstrated the feasibility of integrating CR into augmented reality environments for medical education and surgical planning, with educational applications being among the most highly valued use cases.^22^ Similarly, mixed-reality implementations of CR data have shown promise in enhancing spatial understanding in surgical contexts.^23^ These developments suggest that patient-derived CR models may serve as a foundational dataset for future immersive educational platforms.

### Limitations and Future Directions

This study has several limitations that should be considered when interpreting its findings. First, the retrospective, single-center design and relatively small sample size (n = 35) may limit generalizability to other institutions and patient populations. Second, all CR reconstructions were performed using a single vendor’s implementation (Siemens Syngo.via), and reproducibility across alternative software platforms has not been assessed. Third, complete blinding to pathological status was not always achievable, as overt structural distortion may be visually apparent on CR images; this limitation was partially mitigated through randomized, mixed-order case presentation. Fourth, educational utility was assessed exclusively by 2 senior expert observers and was not validated in learner-centered studies involving medical students, residents, or other trainees; accordingly, the conclusions of this study pertain to perceived educational potential as rated by expert neuroimaging specialists, rather than demonstrated educational effectiveness. Fifth, no direct comparison with other visualization methods, such as conventional volume rendering, virtual reality (VR) environments, or 3D anatomical atlases, was performed within this study, as the intent was to establish a reproducibility-focused baseline for MRI-based CR in the neuroanatomical domain, where existing literature already documents virtual reality (VR) and atlas-based approaches. This study is therefore positioned as a foundational feasibility and perceptual assessment, intended to inform and motivate future learner-centered and comparative investigations.

Future research should focus on learner-centered outcomes, including randomized studies comparing cinematic rendering-based instruction with conventional imaging in neuroanatomy education. Objective assessments of spatial reasoning, knowledge retention, and long-term educational impact will be necessary to define its true pedagogical value. Comparative studies with other digital modalities such as virtual reality and 3D anatomical atlases are also warranted. Furthermore, integration of cinematic rendering into blended learning frameworks combining digital and traditional anatomy teaching may provide additional educational benefits.^24^ Emerging applications in augmented and mixed reality environments^22^^,^^23^ and potential integration with artificial intelligence-based visualization systems represent promising directions for future development. These advancements may further enhance the role of cinematic rendering as a patient-derived, accessible, and scalable resource within modern neuroanatomy education.

## Figures and Tables

**Table 1.** Demographic and Clinical Characteristics of the Study Cohort

Characteristic	Healthy Controls (n = 15)	Brain Tumor (n = 20)	Total (n = 35)
Age, years (mean ± SD)	35.6 ± 17.85	38.8 ± 15.35	37.4 ± 16.3
Age range, years	18-85	17-76	17-85
Female, n (%)	9 (60.0)	9 (45.0)	18 (51.4)
Male, n (%)	6 (40.0)	11 (55.0)	17 (48.6)
Tumor histopathology			
DNET	–	4	
Oligodendroglioma	–	4	
Astrocytoma	–	4	
Glioblastoma	–	4	
Meningioma	–	4	
Tumor location			
Temporal	–	10	
Frontal	–	5	
Parietal	–	4	
Occipital	–	1	
Tumor size range (mm) 3 orthogonal diameters	–	12 × 10 × 11 to 67 × 63 × 45	

Summary of participant data including age, sex, tumor histopathology, anatomical location, and orthogonal size ranges. The even distribution across 5 histopathological subtypes was intentional, enabling assessment of CR educational utility across a biologically diverse spectrum of brain pathologies. Tumor size is reported as 3 orthogonal diameters (length × width × height).

DNET, dysembryoplastic neuroepithelial tumor.

**Table 2.** Scoring Rubric for Visual Assessment Domains

Domain	1 = Poor	2 = Moderate	3 = Good
Image resolution	Blurring or noise markedly reduces clarity	Adequate clarity; minor artifacts present	Sharp, artifact-free reconstruction
Anatomical detail	Key landmarks not identifiable	Most landmarks visible; some ambiguity	All major landmarks clearly depicted
Lesion depiction (tumor cases only)	Tumor-parenchyma interface unclear	Interface partially defined	Tumor boundaries and spatial extent clearly shown
Visualization utility	3D representation adds no spatial information	Partial spatial benefit over 2D imaging	Clear added value for spatial understanding
Educational value	Not suitable for teaching purposes	Limited instructional utility	Suitable as a high-quality educational resource

Criteria used by both independent observers to assign 3-point Likert ratings across all 5 evaluation domains.

3D, 3-dimensional; 2D, 2-dimensional.

**Table 3.** Visual Quality and Educational Value Scores for 3D T1-weighted Cinematic Rendering (CR)

Domain	Healthy Controls (n = 15) Median [IQR]	Brain Tumor (n = 20) Median [IQR]	*P*
**Observer 1**			
Image resolution	3 [3-3]	3 [3-3]	.419
Anatomical detail	3 [2.5-3]	3 [2-3]	.619
Visualization utility	3 [3-3]	3 [3-3]	.419
Educational value	3 [3-3]	3 [3-3]	.229
**Observer 2**			
Image resolution	3 [3-3]	3 [3-3]	.229
Anatomical detail	3 [2.5-3]	3 [2-3]	.619
Visualization utility	3 [2-3]	3 [3-3]	.868
Educational value	3 [3-3]	3 [3-3]	.419

Comparison of healthy controls versus brain tumor patients across 5 evaluation domains. Data are presented as Median [Interquartile Range]. *P*-values refer to independent assessments by 2 senior observers using the Mann–Whitney *U-*test. Lesion depiction was assessed exclusively in tumor cases (n = 20).

Ratings assigned using a 3-point Likert-type scale (1 = poor, 2 = moderate, 3 = good). Data presented as median [interquartile range]. *P*-values calculated using the 2-tailed Mann–Whitney *U-*test.

**Table 4.** Interobserver Agreement for CR Visual and Educational Domains

Domain	n	Observer 1 Median [IQR]	Observer 2 Median [IQR]	Weighted *κ* (95% CI)	Percent Agreement (%)
Image resolution	35	3 [3-3]	3 [3-3]	0.65 (0.00-1.00)	97.1
Anatomical detail	35	3 [2-3]	3 [2-3]	0.87 (0.64-1.00)	94.3
Lesion depiction*	20	3 [2.75-3]	3 [2.75-3]	1.00 (1.00-1.00)	100.0
Visualization utility	35	3 [3-3]	3 [3-3]	0.65 (0.00-1.00)	97.1
Educational value	35	3 [3-3]	3 [3-3]	0.65 (0.00-1.00)	97.1

Reliability metrics expressed as quadratic weighted Cohen’s *κ* values with 95% confidence intervals (1000 bootstrap iterations) and absolute percent agreement rates.

*κ, *quadratic weighted Cohen’s kappa; CI, confidence interval derived from 1000 bootstrap iterations.

^*^Lesion depiction evaluated in tumor cases only (n = 20).

## Data Availability

The data that support the findings of this study are available on request from the corresponding author.
